# Insights into therapeutic resistance from whole-genome analyses of circulating tumor DNA

**DOI:** 10.18632/oncotarget.1486

**Published:** 2013-10-08

**Authors:** Luis A. Diaz, Mark Sausen, George A. Fisher, Victor E. Velculescu

**Affiliations:** Johns Hopkins Kimmel Cancer Center, Baltimore, MD, USA; Personal Genome Diagnostics, Inc., Baltimore, MD, USA; Stanford University Medical Center, Stanford, CA, USA; Johns Hopkins Kimmel Cancer Center, Baltimore, MD, USA

**Figure 1:**
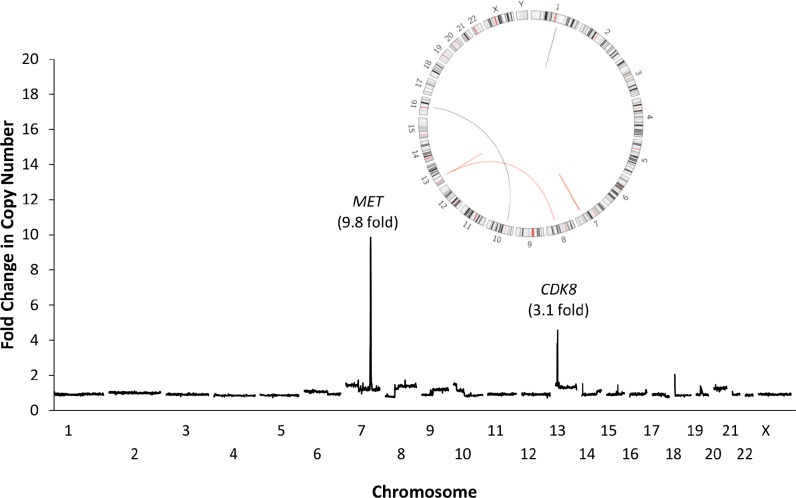
Identification of *MET* amplification through analyses of plasma DNA

The selection and expansion of tumor cells with de novo genetic alterations in specific genes has been described as a mechanism of resistance to targeted therapy. Unfortunately, in the metastatic setting it is often not possible to examine the multiple lesions present in an individual to determine the presence and mechanism of acquired resistance during therapy. Circulating tumor DNA (ctDNA) can be used to analyze the genomic characteristics of tumors in a non-invasive manner [1]. We have previously reported that tumor-specific chromosomal changes can be detected in plasma from patients through integrated whole-genome analyses of cell-free DNA [1]. We have now applied whole-genome sequencing to analyze plasma ctDNA from a patient with chemotherapy-refractory colorectal cancer that had become resistant to EGFR-blockade with cetuximab. A total of 630 Gb of sequence data were obtained, corresponding to 145x sequence coverage of cell-free genomic DNA, and analyzed to identify sequence and structural alterations. These and other analyses revealed emergence of a Q61H mutation in KRAS as well as focal high-level (>9 fold) amplification and rearrangement of the *MET* locus that were not detected in pre-treatment tumor samples (Figure 1). Alterations in these genes have been reported in tumors with secondary resistance to EGFR-directed therapy and are consistent with clinical progression [2-5]. In addition to its role in resistance to targeted therapy, MET amplification has been shown to confer sensitivity to the tyrosine kinase inhibitor crizotinib and other inhibitors [6, 7], and may provide a new therapeutic target in patients with resistance to EGFR blockade. These results describe the first whole-genome analysis of ctDNA to identify genetic alterations associated with mechanisms of resistance to a targeted therapy. Our analyses suggest that genome-wide analyses of ctDNA can be used for discovery of tumor-specific alterations in the in the context of disease monitoring, detection of molecular resistance, and identification of new therapeutic targets.

The amplification of each gene is indicated as fold copy gain, along with the genomic rearrangements detected through whole-genome analyses of cell-free DNA obtained from plasma. Rearrangements associated with focal copy number alterations, including amplification of *MET*, are highlighted in red.
